# Microbial Insight into a Pilot-Scale Enhanced Two-Stage High-Solid Anaerobic Digestion System Treating Waste Activated Sludge

**DOI:** 10.3390/ijerph14121483

**Published:** 2017-11-30

**Authors:** Jing Wu, Zhiping Cao, Yuying Hu, Xiaolu Wang, Guangqi Wang, Jiane Zuo, Kaijun Wang, Yi Qian

**Affiliations:** 1 State Key Joint Laboratory of Environment Simulation and Pollution Control, School of Environment, Tsinghua University, Beijing 100084, China; caozhiping2011@163.com (Z.C.); hu_yuying@foxmail.com (Y.H.); xiaolu_rae@163.com (X.W.); wgq917@163.com (G.W.); jiane.zuo@tsinghua.edu.cn (J.Z.); wkj@tsinghua.edu.cn (K.W.); qiany@tsinghua.edu.cn (Y.Q.); 2 China Northwest Architecture Design and Research Institute Co. Ltd., Xi’an 710018, China

**Keywords:** enhanced two-stage high-solid anaerobic digestion, hydrogenotrophic methanogenesis, syntrophic acetate oxidation, waste activated sludge

## Abstract

High solid anaerobic digestion (HSAD) is a rapidly developed anaerobic digestion technique for treating municipal sludge, and has been widely used in Europe and Asia. Recently, the enhanced HSAD process with thermal treatment showed its advantages in both methane production and VS reduction. However, the understanding of the microbial community is still poor. This study investigated microbial communities in a pilot enhanced two-stage HSAD system that degraded waste activated sludge at 9% solid content. The system employed process “thermal pre-treatment (TPT) at 70 °C, thermophilic anaerobic digestion (TAD), and mesophilic anaerobic digestion (MAD)”. Hydrogenotrophic methanogens *Methanothermobacter* spp. dominated the system with relative abundance up to about 100% in both TAD and MAD. Syntrophic acetate oxidation (SAO) bacteria were discovered in TAD, and they converted acetate into H_2_ and CO_2_ to support hydrogenotrophic methanogenesis. The microbial composition and conversion route of this system are derived from the high solid content and protein content in raw sludge, as well as the operational conditions. This study could facilitate the understanding of the enhanced HSAD process, and is of academic and industrial importance.

## 1. Introduction

Sludge is inevitably generated during wastewater treatment, and has become a pressing global environmental issue. The annual yield of sludge (solid content of 20%) has reached 30 million tons in China [1] and 54.8 million tons in the European Union [2]. Furthermore, sludge contains a high proportion of organic matter, which is apt to decay and consequently attract pathogens and disease vectors [3].

HSAD has become one of the mainstream techniques for the AD of sludge, with the obvious advantages of a small digester, low heating energy, and some amount of digestate [4]. Currently, some full-scale sludge HSAD plants with a solid content of 8–15% are successfully operated worldwide. Thermal treatment could enhance sludge’s anaerobic digestion. For instance, in Chen’s study [5], thermal treatment was proved to degrade macromolecular organic components and thus was beneficial for VS removal. Besides, CH_4_ production, VS removal, and sludge stabilisation of continuous full-scale thermophilic anaerobic digestion of wastewater sludge were also enhanced by the thermal pretreatment in Han’s study [6]. Thermal treatment processes under various temperature ranges have been developed rapidly. For example, it has become the mainstream HSAD technique in England and China. It was estimated that over 80% of anaerobically digested sludge was treated by enhanced HSAD with thermal treatment in China. The enhanced HSAD process with thermal treatment showed its advantages in both methane production and VS reduction.

Microbes are the core workers of the AD process and carry out four successive biological steps, namely hydrolysis, acidogenesis, acetogenesis, and methanogenesis [7]. Fujishima [8] investigated the variation of some microbes in a mesophilic AD digester at a solid content of 3–11% using the most probable number method. Liu [9] demonstrated the variation in the microbial community in a single-stage mesophilic AD reactor when the solid content of sludge increased from 10% to 19%. The major findings were as follows: (1) relative abundance of acetolactic methanogens decreased while relative abundances of hydrogenotrophic methanogens and methylotrophic methanogens increased in the archaeal community; (2) bacteria affiliated to the phylum *Firmicutes* decreased while those of the phylum *Bacteroidetes* increased as the solid content augmented. Wang [10] reported that in a two-phase sludge HSAD system, the microbial compositions of the two phases differed significantly from each other. Thus far, microbial insight into the enhanced HSAD process of sludge is still very limited.

Syntrophic acetate oxidation (SAO) bacteria can oxidize acetate to H_2_ and CO_2_. The H_2_ and CO_2_ are then converted to CH_4_ through a hydrogenotrophic pathway [4]. The presence of SAO bacteria was very important for HSAD, and it was probably a consequence of the inhibitive effect of ammonia on the activity of acetolactic methanogens. Xu [11] demonstrated that SAO bacteria were very important for solid-state anaerobic digestion of cellulosic biomass. Maria [12] suggested that in the liquid digestate of the solid anaerobic digestion, a methanogenic pathway characterized by a relevant syntrophic acetate-oxidizing metabolism was observed.

In previous studies, various factors which significantly affect the microbial composition of conventional AD and HSAD have been investigated, including substrate composition [13,14], ammonia concentration [15,16], OLR [17], reaction temperature [14], and stage and/or phase separation [18]. However, to the best of our knowledge, relative microbial study on enhanced two-stage sludge HSAD systems is still limited.

In order to investigate the relative microbial information in this study, two-stage sludge HSAD systems (including thermophilic anaerobic digestion and mesophilic anaerobic digestion) were conducted after thermal pre-treatment. Then, the microbial community of a pilot-scale enhanced two-stage sludge HSAD system at a steady state was investigated.

## 2. Materials and Methods

### 2.1. Experimental Set-Up

The pilot-scale experiment was conducted over nine months in a wastewater treatment plant in Southern China. The system employed an enhanced two-stage HSAD process. Feed sludge was pumped into the thermal pre-treatment (TPT) reactor (70 ± 1 °C, SRT of 3 days), and was then sent to the two-stage AD (thermophilic anaerobic digestion (TAD, 55 ± 1 °C, SRT of 12.5 days) and mesophilic anaerobic digestion (MAD, 35 ± 1 °C, SRT of 12.5 days) reactors sequentially. The thermal treatment was conducted at 70 °C, because it was economical not only for the low heating energy, but also low investment. Additionally, thermally treating at 70 °C was one of the pasteurisation processes that meets the requirement of Class A solids [3]. The TPT, TAD, and MAD reactors had working volumes of 0.24 m^3^, 1 m^3^, and 1 m^3^, respectively, and were continuously mixed by helical ribbon stirrers. The detailed information of parameters selected for this system was described in our previous study [19].

### 2.2. Inoculum and Feed

The TAD and MAD reactors were inoculated with the same sludge from a full-scale mesophilic sludge anaerobic digester. The inoculum consisted of total solid (TS) of 29.8 g/L and VS of 17.0 g/L. The feed sludge was raw dewatered waste activated sludge (WAS) collected from the wastewater treatment plant where the pilot experiment was conducted. Some characteristics of the raw dewatered WAS are listed in Table 1.

### 2.3. Analytical Methods

In order to evaluate the performance of the pilot HSAD system, the following parameters were regularly monitored. Biogas production and the methane content of biogas were measured daily using a wet gas flowmeter (LMF-2, Changchun Automotive Filter Co. Ltd., Changchun, China) and infrared methane detector (DR95C-CH4-IR, WOST, Shengzhen, China), respectively. A pH meter (PHS-3C, INESA, Shanghai, China) was used to determine the pH. Solid content and VS were measured using the gravimetric method (MEP of China, [20]). Sludge samples were centrifuged at 5000 rpm for 10 min (CR22G, Hitachi, Japan), then the supernatant was filtered through a 0.45 μm filter membrane, and finally, the filtrates were utilised to measure the soluble chemical oxygen demand (SCOD), total ammonia nitrogen (TAN), and total alkalinity (TALK) using standard methods (MEP of China, [20]), i.e., SCOD was tested with the fast digestion-spectrophotometric method using a rapid COD meter (5B-1B, Lianhua Technology Co., Ltd., Lanzhou, China); TALK was determined using the bromocresol green-methyl red indicator titration method; and TAN was measured with Nessler’s reagent spectrophotometric method. Free ammonia nitrogen (FAN) was calculated using the equation provided by Hansen [21].

### 2.4. Microbial Community Analysis

Three sludge samples (each approximately 500 mL) were collected from the TPT, TAD, and MAD reactors at a steady state. Subsequently, 500 μL sludge was taken from each well-mixed sample and washed thrice with phosphate-buffered saline. The buffer was removed as the supernatant after centrifugation (CR22G, Hitachi, Tokyo, Japan) at 10,000 rpm for 10 min. Finally, the sludge pellets were stored at −80 °C before DNA extraction.

The total genomic DNA of each sludge sample was extracted using the FastDNA SPIN Kit for Soil (MP Biomedicals, LLC, California, CA, USA). Bacterial and archaeal 16S rRNA genes were amplified by the polymerase chain reaction (PCR) as described by Wang [22], using the primer pairs 63F (5′-CAGGCCTAACACATGCAAGTC-3′) and 1387R (5′-GGGCGGWGTGTACAAGGC-3′) for bacteria [23], and primer pairs 109F (5′-ACKGCTCAGTAACACGT-3′) and 915R (5′-GTGCTCCCCCGCCAATTCCT-3′) for archaea [24]. The PCR products were purified with a QIAquick PCR purification kit (Qiagen, Hilden, Germany) and then cloned into pGEM-T Easy Vector (Promega Corp., Madison, MI, USA). The clones were sequenced at Shenggong Bioengineering Co., Ltd. (Shanghai, China) using a 3730XL DNA Analyzer (Applied Biosystems, Carlsbad, NM, USA).

The sequences were analysed using QIIME [25]. First, chimeric sequences were identified and removed using the blast_fragments method. Next, the valid sequences were clustered into operational taxonomic units (OTU) at a similarity threshold of 97%. The Shannon rarefaction curve and α-diversity for each sample, as well as the β-diversity of bacterial and archaeal libraries, were calculated on the basis of OTU assignment. Finally, one representative sequence for each OTU was selected and assigned the taxonomy against the Greengenes database.

Bacterial and archaeal neighbour-joining trees were constructed with MEGA5.10 [26] using the Jukes-Cantor algorithm and 1000 bootstrap replicates.

## 3. Results and Discussion

### 3.1. Performance of Pilot HSAD System

The system showed a stable performance after 56 days of operation (i.e., two SRTs) in terms of the biogas production rate, methane content, and key digestate characteristics. Some data of the system at a steady state were listed in Table 1.

At a steady state, the pilot-scale system performed satisfactorily with a total VS removal rate of 44.0% and a methane yield of 0.525 m^3^/kg VS_removed_. The methane content of biogas was 65.3 ± 1.9%. TPT, TAD, and MAD contributed to VS reduction at proportions of 36.2%, 44.8%, and 19.0%, respectively.

Table 1 also revealed that the concentrations of SCOD, TALK, and TAN in the TPT reactor were significantly higher than those of raw sludge. This indicates that some particle organic matter was converted into soluble organics in the TPT reactor, which would be more easily utilised by microbes. Most soluble organics were then degraded in the subsequent two-stage HSAD process. This led to a dramatic decrease in SCOD concentration in both TAD and MAD reactors.

The pilot-scale enhanced two-stage HSAD system converted some sludge organics into biogas and reduced sludge at a high solid content. The high performance of the system might be the result of two reasons. Firstly, TPT might enhance the hydrolysis of sludge to increase the SCOD concentration, and consequently improve the VS removal rate and biogas yield. As was widely accepted, hydrolysis was the rate-limiting step of sludge AD. Several studies have already demonstrated the enhancement of thermal pre-treatment on the anaerobic digestion of sludge with a solid content of 2.5–15% [27,28,29]. Secondly, the two-stage digestion process (i.e., TAD-MAD) might be beneficial in this HSAD system. As demonstrated by Azbar and Speece [30], the subtle differences in environmental conditions (e.g., pH, temperature) and the concentration gradient of various substrates in a staged system could lead different predominant microbes to grow in different stages and consequently to improve the anaerobic digestion efficiency and stability.

### 3.2. Microbial Community Structures

**Figure 1:**
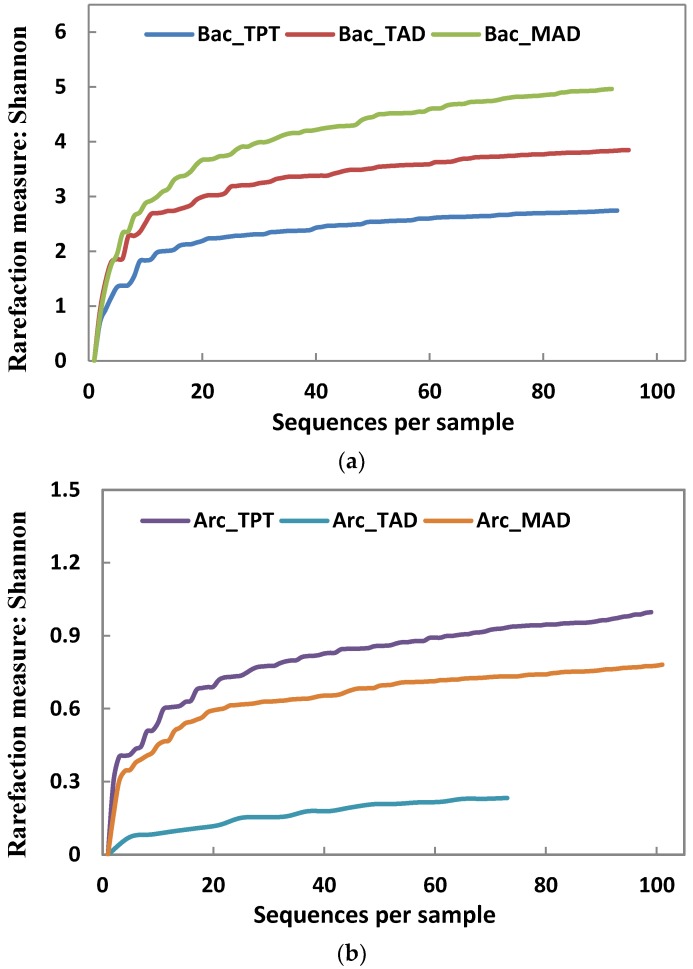
Shannon rarefaction curves of (**a**) bacterial and (**b**) archaeal clone libraries.

A Shannon rarefaction curve (Figure 1) was employed to test the availability of the clone library. Figure 1 showed that the Shannon rarefaction curve of each clone library flattens as the number of valid sequences increases. This indicated that the sequences in each library were large enough to cover most of the microbial information and thus the relative analysis is reliable.

Some critical structural parameters of the clone libraries were presented in Table 2. As indicated by α-diversity, including Chao1 and the Shannon index, sludge samples were significantly different from each other in terms of richness and diversity for both bacterial and archaeal libraries. This probably originated from different substrates and environmental conditions in each phase and stage. The bacterial community diversity ascended from TPT to TAD and finally to MAD. This could be because the fraction of recalcitrant and complex components increased as the readily biodegradable substrates were degraded. Degradation of recalcitrant components required more diverse bacteria. In contrast, the archaeal community diversity decreased from TPT to TAD and then increased from TAD to MAD, showing a negative correlation with FAN concentration. This might be because high concentrations of FAN might strongly inhibit acetoclastic *Methanosaeta* spp. and *Methanosarcina* spp. [31].

The β-diversity of microbial communities showed dissimilarity among samples (Table 3). Generally, dissimilarities in bacterial libraries were higher than those in archaeal libraries, and the dissimilarities of the pairs of TPT and MAD reactors were highest for both archaeal and bacterial communities. This was probably due to the remarkably different reaction temperatures and substrates present in the TPT and MAD reactors.

### 3.3. Composition of Microbial Communities

#### 3.3.1. Composition of Bacterial Communities

**Figure 2:**
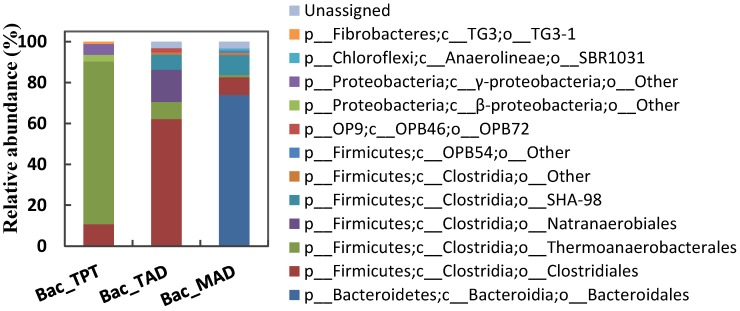
Taxonomic classification of bacterial communities (at the order level).

**Figure 3:**
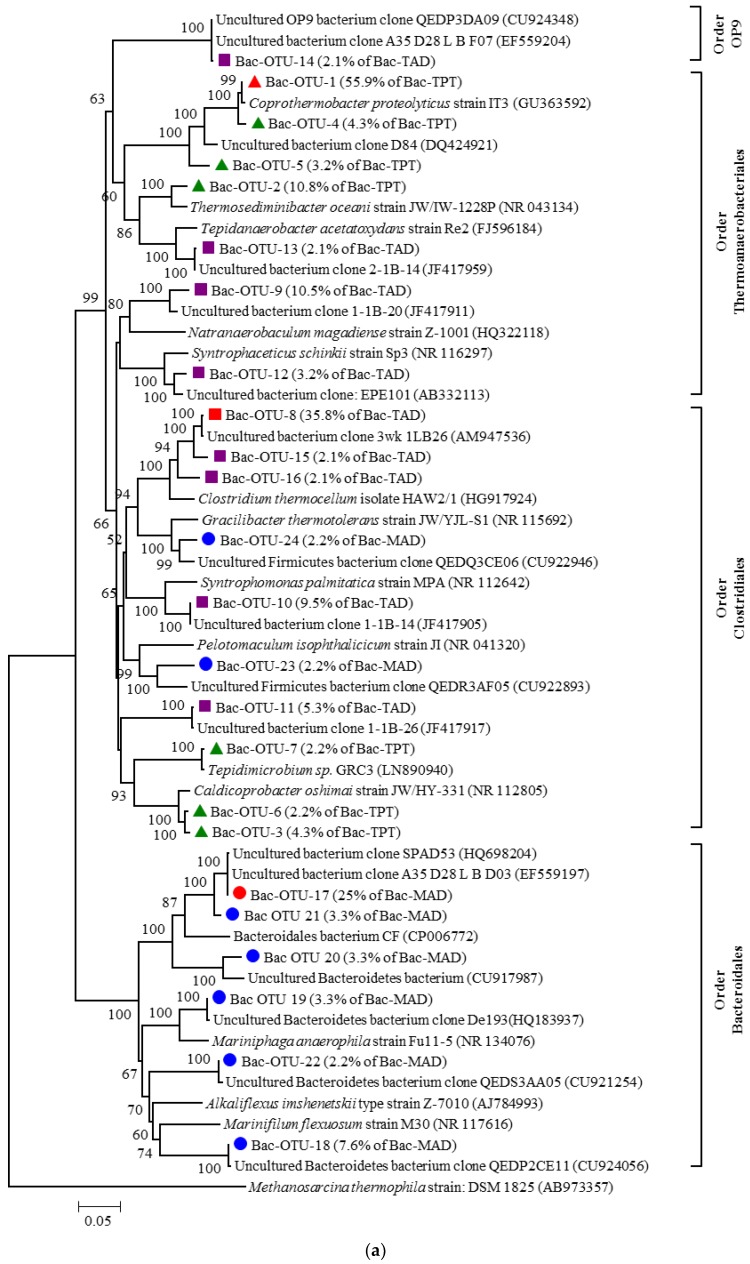

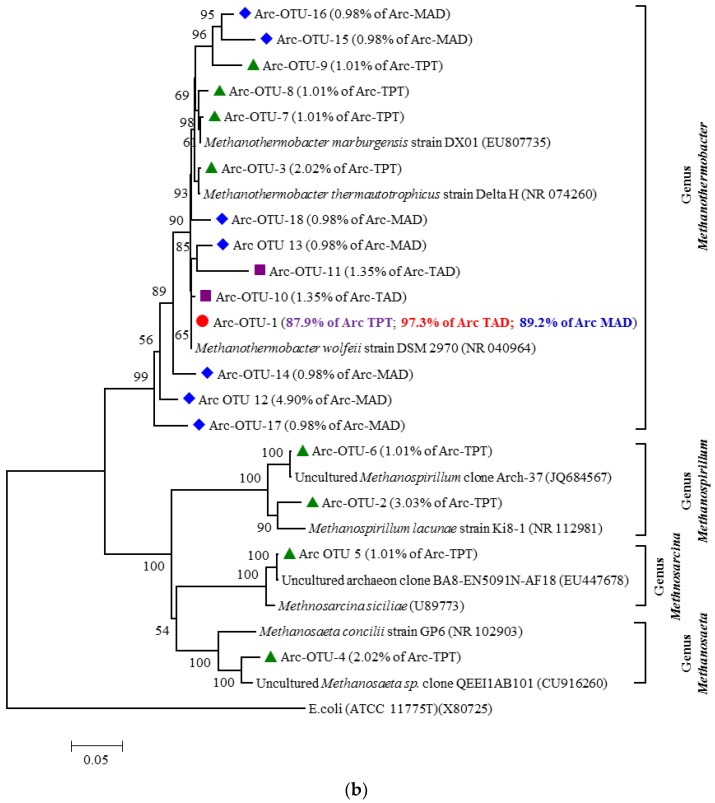
Neighbour-joining phylogenetic trees of (**a**) bacterial and (**b**) archaeal communities.

As the hydrolysis and acidogenesis of anaerobic digestion were carried out by various bacteria, the bacterial communities of each reactor (i.e., TPT, TAD, and MAD) in the pilot-scale sludge HSAD system were of high richness and diversity (Table 2). They differed from each other with respect to composition at the order level (Figure 2). The predominant bacterial orders in TPT, TAD, and MAD reactors were *Thermoanaerobacteriales*, *Clostridiales*, and *Bacteroidales*, respectively, with relative abundances of 79.8%, 62.1%, and 73.9% in corresponding clone libraries. This indicated that the bioprocesses in these three reactors were distinct from each other. More information about the conversions was available from the neighbour-joining phylogenetic tree (Figure 3a), which was constructed using the main OTUs (containing two or more sequences in at least one sample). Relatives in the phylogenetic tree shared similar physiological features and functions.

##### Composition of Bac-TPT Library

As shown in Figure 3a, in the bacterial library of TPT (named Bac-TPT library), the predominant Bac-OTU-1 (with relative abundance of 55.9%) and Bac-OTU-4 and 5 (with relative abundances of 4.3% and 3.2%, respectively) were closely related to thermophilic *Coprothermobacter proteolyticus* strain IT3 (GU363592). *C. proteolyticus* could degrade proteins and amino acids with a high hydrogen production capacity [32]. *Coprothermobacter* spp. has been found in several thermophilic anaerobic sludge digesters [33,34,35] and their relative abundance also reached 23% in the thermophilic acidification reactor of a two-phase anaerobic digestion system [33]. In our pilot system, the high reaction temperature and phased conditions in TPT promoted the growth of proteolytic *Coprothermobacter* spp., which dominated in the Bac-TPT library with a total relative abundance of 63.4%. Proteolysis was very important for this HSAD system because protein, as the major organic component, accounted for 53.6% of organic matter in the feed sludge. It decreased from 30.2 g/L in raw WAS to 20.6 g/L in TPT sludge with a reduction rate of approximately 30%. Moreover, abundant hydrogen and ammonia produced by proteolysis played an important role in the subsequent two-stage anaerobic digestion (i.e., TAD-MAD) because the former was directly utilised by hydrogenotrophic methanogens as a substrate, while the increased concentration of the latter (from 522 mg/L in raw sludge to 1858 mg/L in TPT sludge) inhibited the growth of acetoclastic methanogens [31].

There were some common physiological features among the relatives of Bac-OTU-2, 3, 6, and 7 of the Bac-TPT library in the phylogenetic tree (Figure 3a), including the utilisation of various monosaccharides and oligosaccharides to produce acetate or CO_2_ and H_2_; existence in thermophilic conditions with an optimum temperature of around 70 °C; and tolerance of high salinity ranging from 0% to 6% [36,37]. In addition, a relative of Bac-OTU-2 (with relative abundance of 10.8%), *Thermosediminibacter oceani* strain JW/IW-1228P (NR_043134), could utilise casamino acids, tryptone, pyruvate, and methanol [36]. A close relative of Bac-OTU-7 (with relative abundance of 2.2%), *Tepidimicrobium* sp. GRC3 (LN890940), could degrade cellulose, a typical recalcitrant polysaccharide.

The above-mentioned bacterial composition indicated that it was possible that some proteins, easily-biodegradable saccharides, peptones, and even some recalcitrant cellulose might be decomposed in the TPT reactor. This agreed with the fact that the concentrations of SCOD and TAN in TPT increased significantly compared to those of raw sludge.

##### Composition of Bac-TAD Library

The most abundant bacteria in Bac-TAD were halotolerant or ammonia-tolerant degraders of cellulose and other simple carbohydrates. As shown in Figure 3a, Bac-OTU-8, 15, and 16 (with a total relative abundance of 40.0%) were closely related to the uncultured clone 3wk_1LB26 (AM947536) and cultured *Clostridium thermocellum* isolate HAW2/1 (HG917924) with a sequence similarity higher than 95%. The uncultured clone 3wk_1LB26 was retrieved from a thermophilic anaerobic sludge digester with high levels of salt (20 g/L) and ammonia (2.7 g/L) [38]. *C. thermocellum* HAW2/1 degraded cellulose and cellobiose to produce acetate and other products at 55 °C [39]. After prolonged incubation, some isolates of *C. thermocellum* HAW2/1 could also utilise glucose, fructose, or sorbitol [39]. Generally, cellulose was regarded as a recalcitrant organic. The presence of abundant cellulolytic bacteria indicated the possibility of cellulose degrading in the TAD reactor.

The second largest cluster in the Bac-TAD library was Bac-OTU-10, with a relative abundance of 9.5%. Its closest relative was the uncultured bacterium clone 1-1B-14 (JF417905) isolated from a dry thermophilic methanogenic digester treating artificial waste paper [40]. With respect to previously characterized relatives, this OTU was also closely related to *Syntrophomonas palmitatica* strain MPA (NR112642), with a sequence similarity of 93%. *S. palmitatica* MPA could utilise straight-chain saturated fatty acids with four to 18 carbon atoms to produce acetate and/or propionate when co-cultured with the hydrogenotrophic methanogen *Methanospirillum hungatei* [41]. These bacteria were important for this system, because long-chain fatty acids (LCFAs) could acutely inhibit anaerobic consortia (especially methanogens) by adsorption onto cell walls or membranes, and interference with transport or protective functions, consequently hampering the anaerobic digestion process [42].

As shown in Figure 3a, Bac-OTU-12 and 13 (with relative abundances of 4.2% and 2.1% respectively) shared a 95% sequence similarity with *Syntrophaceticus schinkii* strain Sp3 (NR_116297) [43] and *Tepidanaerobacter acetatoxydans* strain Re2 (FJ596184) [44]. Both *S. schinkii* Sp3 and *T. acetatoxydans* Re2a were syntrophic acetate oxidation (SAO) bacteria that thrived in high NH_4_Cl concentrations (up to 6 g/L). SAO bacteria could oxidize acetate to H_2_ and CO_2_, which was then converted to CH_4_ through a hydrogenotrophic pathway. Schnürer [45] reported that SAO bacteria benefit from the high temperatures and high ammonia concentrations derived from the degradation of protein-rich material. Several studies indicated that SAO is the dominant pathway of acetate methanogenesis when acetolactic methanogens were inhibited by high ammonia nitrogen [31,46]. Westerholm [16] demonstrated that SAO bacteria were very important for mesophilic methanogenic systems with high ammonia concentrations, as their absence would cause system deterioration. In our pilot system, the high ammonia nitrogen concentration derived from the decomposition of protein and high temperatures in TAD benefited the growth of SAO bacteria.

Thermodynamically, acetate oxidation was unfavourable under standard conditions (CH_3_COO^−^ + 4H_2_O → 4H_2_ + 2HCO_3_^−^ + H^+^; ΔG^0′^ = +105 kJ) and could only proceed via syntrophic interaction between some acetate-oxidising bacteria and hydrogenotrophic methanogens, as hydrogenotrophic methanogens kept the H_2_ partial pressure low. Thus, this pathway was named syntrophic acetate oxidation. The SAO pathway has been directly proved by isotopic carbon analyses [15,46] and has been observed in various anaerobic digesters [15,16,45,47,48]. Until now, only a few SAO bacteria had been reported including *Clostridium ultunense*, *S. schinkii*, *T. acetatoxydans*, *Thermacetogenium phaeum*, *Thermotoga lettingae* [16], and cluster II Spirochaetes [49].

In the phylogenetic tree (Figure 3a), the Bac-OTU-9, 11, and 14 of the Bac-TAD library shared less than an 88% sequence similarity with any cultured relative. However, they all had uncultured close relatives with more than a 97% sequence similarity. This indicated that the bacteria retrieved from the present system were prevalent although not well understood. Interestingly, some of the uncultured relatives of Bac-OTU-9 and Bac-OTU-11, e.g., clone 1-1B-20 (JF417911) and clone 1-1B-26 (JF417917), were retrieved from the same dry thermophilic anaerobic digester used to degrade cellulose [40]. Bac-OTU-14 (with relative abundance of 2.1%) shared a 99% sequence similarity with the uncultured bacterium clone A35_D28_L_B_F07 (EF559204), which was identified to be associated with acetate methanization through the SAO pathway with the help of the DNA-stable isotope probing technique [50]. Additionally, Bac-OTU-14 also had uncultured relatives from sludge anaerobic digesters, e.g., the uncultured clone QEDP 3DA09 (CU924348) [13].

The bacterial composition in the TAD reactor showed that cellulose and some other simple carbohydrates and LCFAs could be utilised in this reactor. SAO bacteria can convert acetate into H_2_ and CO_2_ to feed hydrogenotrophic methanogens. This provided an alternative for acetate methanogenesis besides the acetoclastic methanogenesis pathway.

##### Composition of Bac-MAD Library

As the majority of readily biodegradable organics are degraded in TPT and TAD, most organics in MAD were recalcitrant organic matter or intermediate products. As a result, the Bac-MAD library exhibited a relatively high richness and diversity (Table 2). There were 92 clones in the Bac-MAD library, which were assigned to 55 OTUs. The close relatives of bacteria retrieved in the MAD reactor with a sequence similarity higher than 94% were prevalent in various anaerobic artificial reactors or natural environments. However, it was difficult to understand the bioprocesses in this reactor because only 7.6% of these clone sequences had definite taxonomy assignment at the genus level; even at the family level, only 20.7% had definite taxonomic assignment. Only 48.9% of clones were included in the tree because only OTUs greater than 1 were included.

Almost all OTUs of the Bac-MAD library in the bacterial phylogenetic tree (Figure 3a) were affiliated with the order *Bacteroidales* and were significantly different from the bacterial OTUs of the TPT and TAD reactors, except for Bac-OTU-23 and 24, which belonged to the order Clostridiales.

The predominant Bac-OTU-17 (with a relative abundance of 25%) and Bac-OTU-21 (with a relative abundance of 3.3%) constituted the largest group in Bac-MAD. Close neighbours of this group contained uncultured bacterium clone A35_D28_L_B_D03 (EF559197, 100% sequence similarity) and clone SPAD53 (HQ698204, 99% sequence similarity). Clone A35_D28_L_B_D03 was probably another SAO bacterium retrieved from the ^13^C-acetate methanogenesis system [50]. Clone SPAD53 was retrieved from a keratin (recalcitrant organic material) digestion system. In the system of Xia [51], a TAN concentration of 5.5–7.5 g/L was beneficial to the SAO pathway. However, it was difficult to precisely identify the family and genus categories of both Bac-OTU-17 and Bac-OTU-21 due to the lack of cultured relatives.

#### 3.3.2. Composition of Archaeal Communities

The archaeal phylogenetic tree (Figure 3b) of TPT, TAD, and MAD was constructed with all OTUs in the archaeal libraries. As shown in the tree, all archaeal clones retrieved from this system were assigned to only four genera: *Methanothermobacter*, *Methanospirillum*, *Methanosarcina*, and *Methanosaeta*. Moreover, the genus *Methanothermobacter* dominated all archaeal libraries with relative abundances of 93.0%, 100%, and 100% in Arc-TPT, Arc-TAD, and Arc-MAD libraries, respectively, with a common predominant OTU, Arc-OTU-1 (with relative abundances of 87.9–97.3%). This predominant OTU shared a 100% sequence similarity with *Methanothermobacter wolfeii* strain DSM 2970 (NR 040964) (Figure 3b). The other OTUs affiliated to genus *Methanothermobacter* also shared high sequence similarities (93–99%) with previously characterised *Methanothermobacter* spp. strains. Only a small proportion (7.1%) of clones in Arc-TPT (represented by Arc-OTU-2, 4, 5, and 6) were affiliated to the genera *Methanospirillum*, *Methanosarcina*, and *Methanosaeta*, with sequence similarities of 93%–98% to their cultured relatives.

Of the four methanogen genera in this HSAD system, the genera *Methanothermobacter* and *Methanospirillum* were typical hydrogenotrophic methanogens; the genus *Methanosarcina* could utilise acetate to produce methane but some species of the genus could also utilise H_2_ and CO_2_ for methanogenesis; only the genus *Methanosaeta* was generally regarded as containing strictly acetoclastic methanogens which could directly cleave acetate to form methane [50,52]. The relative abundance of *Methanothermobacter* spp. of TAD reached 100%, and it could be deduced that methanogenesis was predominant by hydrogenotrophic methanogens in this system. This was significantly different from conventional AD systems dominated by acetoclastic methanogens [52]. 

Methanogenesis, the last and most important step of the anaerobic digestion process, was generally accomplished by two groups of archaeal anaerobes: acetoclastic and hydrogenotrophic methanogens. Demirel and Scherer [52] reviewed the activity and behaviour of both methanogenic archaeal groups during the anaerobic conversion of various forms of biomass and concluded that they were heavily influenced by substrates and the operational conditions of digesters. In this study, substrate and operational conditions of the pilot-scale sludge HSAD system were as follows: (1) protein was abundant in raw sludge; (2) there was a high solid content; and (3) TPT-TAD-MAD was a two-phase and two-stage process, in which the hydrolysis phase was thermally enhanced and the principal methanogenesis phase (TAD) was operated at thermophilic and mesophilic conditions with a short SRT of 12.5 days. The extreme dominance of hydrogenotrophic *Methanothermobacter* in this system might be derived for the following reasons.

Firstly, hydrogenotrophic methanogens had a higher tolerance to the high levels of ammonia nitrogen compared to acetoclastic methanogens. The high solid content of protein-rich raw sludge, and the thermophilic and phased conditions of TPT would stimulate the growth of proteolytic *Coprothermobacter* spp. (accounting for 63.4% of clones in Bac-TPT). With the degradation of abundant proteins, the ammonium concentration increased inevitably. A high level of ammonia might inhibit methanogenesis [46]. Additionally, inhibition by ammonia was more pronounced in thermophilic anaerobic digesters (like TAD in this system) [42], because the concentration of free ammonia (NH_3_)—a stronger inhibitor than ammonium ion (NH_4_^+^)—would increase with rising temperatures. Many studies of other organic wastes have demonstrated that hydrogenotrophic methanogenesis dominated thermophilic anaerobic digesters with high ammonia concentrations [15,38,40] or even mesophilic digesters with high ammonia concentrations. In the dry thermophilic methanogenic digester studied by Tang [40], 90% of clones were assigned to strictly hydrogenotrophic methanogens, and the remaining clones were affiliated with mixotrophic *Methanosarcina*; no strictly acetoclastic *Methanosaeta* relatives were found.

Secondly, the short SRT of 12.5 days in TAD would benefit the predominance of hydrogenotrophic methanogens due to their short generation time [52].

Furthermore, some SAO bacteria existed in this system and played important roles in the exclusive dominance of hydrogentrophic methanogenesis. The presence of SAO bacteria was probably a consequence of the inhibitive effect of ammonia on the activity of acetoclastic methanogens. The syntrophic oxidation of acetate to H_2_ and CO_2_ benefited this system in two respects: (1) by providing more substrates to hydrogenotrophic methanogens (the partner of SAO bacteria) and (2) by preventing acetate accumulation in the system. In a thermophilic anaerobic digestion system treating organic waste, approximately 80% of acetate was decomposed via the SAO pathway [15]. Wett [48] demonstrated that hydrogenotrophic methanogens accounted for 77% of archaeal clones, and SAO was important in their system, which had a high ammonia concentration.

### 3.4. Possible Conversion Route of Organics of Sludge

All microbial compositions are summarised in Table 4. Different dominant bacteria existed in TPT, TAD, and MAD reactors to degrade different sludge organics. According to the microbial compositions, simple carbohydrates could be decomposed throughout the process: proteolysis might occur mainly in TPT; cellulose degradation might begin in TPT and thrived in TAD; and the decomposition of LCFA might occur only in TAD. Methane was mainly produced in TAD through the hydrogenotrophic methanogenesis pathway conducted by *Methanothermobacter*-like archaea. Acetate could be oxidized to H_2_ and CO_2_ by SAO bacteria, and then utilised by hydrogenotrophic methanogens.

**Figure 4:**
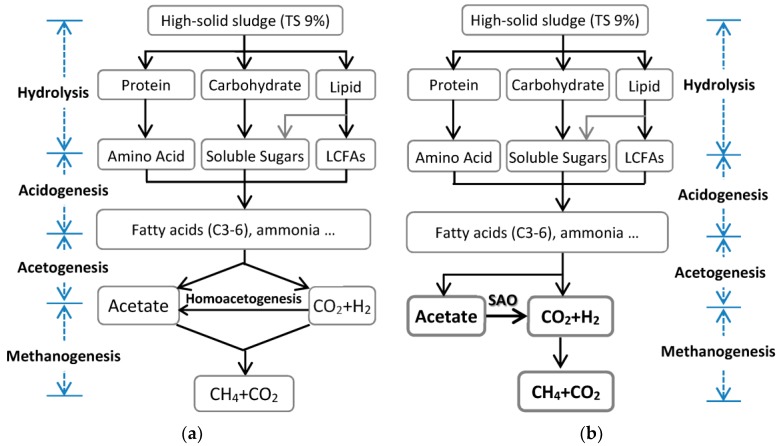
Scheme of (**a**) classic anaerobic digestion theory and (**b**) possible conversion route of sludge to methane in the high-solid anaerobic digestion (HSAD) system.

According to the microbial compositions (Table 4) and the classic theory of anaerobic digestion [53], the possible conversion route of organics in high-solid-content sludge to methane in this pilot system is shown in Figure 4. Hydrogenotrophic methanogenesis was the predominant methanogenesis pathway in this pilot system, rather than acetolactic methanogenesis. This methanogenesis pathway and SAO bacteria played key roles in the system. To the best of our knowledge, no other sludge AD systems have been as predominated by hydrogenotrophic methanogenesis as in our pilot system.

## 4. Conclusions

A two-stage HSAD process enhanced by thermal pre-treatment at 70 °C could effectively reduce and stabilise sludge, and convert some organic matter into methane. The hydrogenotrophic methanogenesis pathway dominated our pilot system. SAO played an important role in the methanogenesis to provide H_2_ and CO_2_ by oxidising acetate. The microbial composition and conversion route of this system were derived from the high solid and protein content in raw sludge, as well as the operational conditions in the phased and staged AD process.

## Figures and Tables

**Table 1:** Characteristics of sludge.

Parameters	Raw WAS	TPT	TAD	MAD
pH	6.1 ± 0.2	6.5 ± 0.0	7.8 ± 0.3	7.6 ± 0.2
Solid content (%)	8.75 ± 0.78	7.90 ± 0.64	6.77 ± 0.40	6.23 ± 0.24
VS (%)	5.64 ± 0.69	4.82 ± 0.38	3.72 ± 0.22	3.21 ± 0.17
VS/TS (%)	64.98 ± 3.02	59.62 ± 2.92	55.00 ± 0.45	51.61 ± 0.98
SCOD (mg/L)	6899 ± 585	16,747 ± 2190	5361 ± 526	1661 ± 342
TALK (mg CaO/L)	479 ± 113	1650 ± 423	1903 ± 300	2476 ± 387
TAN(mg/L)	522 ± 144	1858 ± 163	1058 ± 192	1245 ± 230
FAN (mg/L)	0.4 ± 0.3	51.4 ± 4.5	118.7 ± 21.8	42.7 ± 7.9

All data were obtained during steady state and are given as arithmetic mean ± standard deviation. WAS: waste activated sludge; TPT: thermal pre-treatment; TAD: thermophilic anaerobic digestion; MAD: mesophilic anaerobic digestion.

**Table 2:** Operational taxonomic units (OTUs) and α-diversity indices of bacterial and archaeal clone libraries.

Clone Library	Bacteria			Archaea		
	Bac-TPT	Bac-TAD	Bac-MAD	Arc-TPT	Arc-TAD	Arc-MAD
Clone totality	101	101	101	100	74	102
Chimera count	8	6	9	1	0	0
Valid sequence count	93	95	92	99	74	102
OTU count	23	35	55	9	3	8
α-diversity indices						
Chao1	63	100	325	12	4	23
Shannon index	2.73	3.85	4.96	0.88	0.21	0.75

**Table 3:** β-diversity of microbial communities (weighted).

Bacteria				Archaea			
	Bac-TPT	Bac-TAD	Bac-MAD		Arc-TPT	Arc-TAD	Arc-MAD
Bac-TPT	0			Arc-TPT	0	0.0215	0.0228
Bac-TAD	0.3660	0		Arc-TAD		0	0.0092
Bac-MAD	0.5901	0.5210	0	Arc-MAD			0

**Table 4:** Summary of the main microbial compositions in the HSAD system.

Clone Library		Main Microbes	Relative Abundance	Hydrolysis & Acidogenesis				SAO	Methanogenesis	Unclear
				Protein/Amino Acid	Simple Carbohydrates	Polysaccharide	LCFAs			
**Bacteria**	**Bac-TPT**	*Coprothermobacter*	**63.4%**	**+**						
		*Thermosediminibacter*	10.8%	**+**	**+**					
		*Caldicoprobacter*	6.5%		**+**					
		*Tepidimicrobium*	2.2%		**+**	**+**				
	**Bac-TAD**	*Syntrophaceticus*	4.2%					**+**		
		*Tepidanaerobacter*	2.1%					**+**		
		*Clostridium*	**40.0%**		**+**	**+**				
		*Syntrophomonas*	9.5%				**+**			
		Unclassified 1	2.1%					**+/−**		
		Unclassified 2	15.8%							**+**
	**Bac-MAD**	*Mariniphaga*	3.3%		**+**					
		*Gracilibacter*	2.2%	**+**	**+**					
		Unclassified 1	28.3%					**+/−**		
		Unclassified 2	15.3%							**+**
**Archaea**	**Arc-TAD**	*Methanothermobacter*	**100%**						**+**	
